# Pediatric HIV infection due to maternal transmission: a solvable problem in a peri-urban setting in Bamako, Mali

**DOI:** 10.1186/1742-4690-9-S2-P230

**Published:** 2012-09-13

**Authors:** K Tounkara, A Traore, B Aboubacar, O Koita, B Traore, F Siby Diallo, F Bougoudogo, Y Kone, C Gomez-Mira, J Toffoli, L Levitz, M Rochas, AS De Groot

**Affiliations:** 1 GAIA Vaccine Foundation, Bamako, Mali; 2 Cellule Sectorielle de la Lutte Contre le SIDA, Bamako, Mali; 3 Direction Regionale de la Sante, Bamako, Mali; 4 Institut National pour la Sante Publique, Bamako, Mali

## Background

In 2005, GAIA Vaccine Foundation established a mother-to-child transmission prevention (MTCTP) program (Chez Rosalie) in the community-based clinic of Sikoro, a peri-urban low-resource setting in Bamako, Mali. HIV testing of the pediatric population by PCR was recently implemented. Here we report the status of children born to HIV-seropositive mothers followed at the clinic.

## Methods

The MTCTP program at Chez Rosalie was one of the first such programs to be established. Standard finger-stick approaches to testing children for HIV at 18 months resulted in very few children tested, as women frequently did not return to the clinic with their children at this time. In 2010, PCR-testing of newborns was integrated into mothers’ postpartum follow-up appointments, during which artificial milk and antiretrovirals (ARV) were also distributed as part of PMTCT. As a result of this change, the test has now been performed for 69 children of the 202 HIV-seropositive mothers enrolled in the Chez Rosalie PMTCT program in Sikoro.

## Results

62 children (90%) were HIV-negative by PCR, and seven children (10%) were HIV-positive. Of the seven HIV-positive children, only one was born to a mother followed at the clinic. This mother was diagnosed late in her pregnancy and did not strictly adhere to MTCTP, including exclusive formula feeding her child (the national policy at the time). The other six HIV-positive children were born either at home or in another clinic where MTCTP was not available.

## Conclusion

PCR testing of newborns increased the number of children screened for HIV infection in this MTCTP program. Both treating mothers with ARVs prior to delivery and providing newborns with formula (or exclusive breastfeeding while on ARV) reduced prevalence of pediatric HIV infection by close to 98%. Based on our results, the introduction of MTCTP at the community level is one of the most successful non-vaccine HIV prevention interventions.

